# Proximity Effects of Methyl Group on Ligand Steric Interactions and Colloidal Stability of Palladium Nanoparticles

**DOI:** 10.3389/fchem.2020.00599

**Published:** 2020-07-09

**Authors:** Peter Tieu, Vincent Nguyen, Young-Seok Shon

**Affiliations:** ^1^Department of Chemistry and Biochemistry, California State University, Long Beach, CA, United States; ^2^Keck Energy and Materials Research Program, California State University, Long Beach, CA, United States

**Keywords:** nanoparticles, catalysis, stability, ligands, steric interactions, hydrogenation, isomerization

## Abstract

Metal nanoparticle catalysts functionalized with small, well-defined organic ligands are important because such systems can provide a spatial control in the catalyst-substrate interactions. This article describes the synthesis, stability, and catalytic property evaluations of four different Pd nanoparticles capped with constitutional isomers of pentanethiolate ligands that have either a straight chain or an alkyl chain with one methyl group at different locations (α, β, or γ from the surface-bound sulfur). The structure and composition analyses of Pd nanoparticles confirm that they have similar average core sizes and organic ligand contents. The presence of methyl group at α position is found to lower the capping ability of short ligands and thus make Pd nanoparticles to lose their colloidal stability during the catalytic reactions. The overall activity and selectivity for hydrogenation and isomerization of pentene and allylbenzene derivatives are investigated for each combination of ligand and substrate. Catalysis results indicate that steric interactions between the ligands on the metal catalyst surface and the alkene substrates are a factor in controlling the activity of Pd nanoparticles. In particular, Pd nanoparticles capped with pentanethiolate isomer having a methyl group at α position exhibit poor and inconsistent catalytic activity due to its low colloidal stability. The presence of a methyl group at β position mildly interferes the adsorption of alkene group on the nanoparticle surface resulting in lower conversion yields. Interestingly, a methyl group at γ position only has a minimal effect on the catalytic property of Pd nanoparticles exhibiting similar catalysis results with Pd nanoparticles capped with straight chain pentanethiolate ligands. This indicates the proximity of steric group at the reactive site controls the nanoparticle activity for surface oriented reactions, such as hydrogenation and isomerization of alkenes in addition to their colloidal stability.

## Introduction

Nanoparticles serve as a bridge between atoms and bulk materials, exhibiting a variety of unique chemical, physical, and electronic characteristics (Lohse and Murphy, 2012; Heiligtag and Niederberger, 2013; Henkel et al., 2020). However, the high surface area of small nanoparticles usually decreases the colloidal stability of nanoparticles and causes uncontrolled agglomeration producing thermodynamically favorable large or bulk materials (Ingham et al., 2011; King et al., 2018). Therefore, nanoparticles must be stabilized kinetically through the use of supports, particle encapsulation, or capping ligands (Zhang et al., 2016; Goodman et al., 2017; Kister et al., 2018; Heuer-Jungemann et al., 2019). One of the popular methods in ligand stabilization of nanoparticles is the use of alkanethiol ligands that are known to form a strong covalent bond with various metal nanoparticles (Shon et al., 2008; Elliott and Hutchison, 2015). Alkanethiolate-capped nanoparticles are a well-defined system with high density of structurally organized monolayers (Pensa et al., 2012). The highly passivated surface, however, makes the metal surface of alkanethiolate-capped nanoparticles inefficient for surface catalytic reactions (Campisi et al., 2016; Lu et al., 2018). This has been the reason why only a few examples of alkanethiolate-capped nanoparticles have been successful for organic reactions, such as the Suzuki-Miyaura cross-coupling reaction and the reduction of nitroaromatics, prior to the recent upsurge of publications in the last few years (San and Shon, 2018; Du et al., 2020).

Our research group utilizes the thiosulfate method to assemble alkanethiolate ligands onto metal nanoparticles (Gavia et al., 2014; San et al., 2017; Vargas et al., 2019). The subsequent cleavage of the ionic sulfite moiety after surface adsorption and the presence of hydrophobic monolayer delaying the further passivation of ionic alkyl thiosulfate ligands allow controlling the ligand density on palladium nanoparticles (PdNPs) as described in the previous publications. These straight chain alkanethiolate-capped PdNPs were shown to selectively catalyze the isomerization of allylic alcohols to carbonyl compounds and the mono-hydrogenation of isolated, conjugated, and cumulated dienes in the presence of H_2_ (Zhu and Shon, 2015; Maung et al., 2017; Chen and Shon, 2018). The surface ligand density of alkanethiolate on PdNPs with same average core size was also found to have a great influence on both the activity and selectivity of PdNPs (Vargas et al., 2019). These catalytic reactions take place in homogeneous conditions that normally promote catalytic efficiency and selectivity and complete under the mild condition of room temperature and atmospheric pressure. By introducing various functional groups, such as carboxylic acid and phenyl groups to PdNPs, the previous work has shown that the diverse catalytic platforms for specific chemical interactions could be created (Chen et al., 2017; Maung and Shon, 2017; Mahdaly et al., 2019). The results clearly demonstrated that controlling the partial poisoning of catalytic metal nanoparticle surface with well-defined alkanethiolate ligands bring about the development of highly selective and efficient catalytic materials.

**Figure 1:**
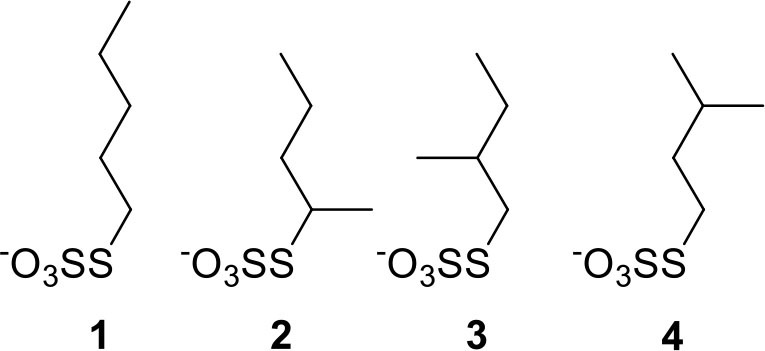
Constitutional isomers of pentyl thiosulfate ligands used for the passivation of Pd nanoparticles.

The unique structural property associated with the presence of alkanethiolate ligands around active catalytic sites of PdNPs enables a molecular approach to these nanoparticles for organic reactions similar to organometallic catalysts (Geukens and de Vos, 2013; Rossi et al., 2018). As an effort to prove the feasibility of controlling the ligand-substrate steric interactions on the reactive surface sites using a methyl branch group and to establish a greater understanding of the role of small ligand structure, this article reports the catalytic activity and selectivity of the PdNPs capped with the constitutional isomers of pentanethiolate during the model catalytic reaction, the hydrogenation/isomerization of substituted terminal alkenes. The competition between hydrogenation and isomerization processes during the catalytic reactions of unsaturated compounds has been one of the main problems associated with the Pd based catalysts including supported Pd or Pd-polymer catalysts) (Gauthier et al., 2010; Zhu and Shon, 2015; Pang et al., 2016). The catalytic activities of PdNPs synthesized from precursor **1** will be compared to those of Pd nanoparticles synthesized from precursors **2**, **3**, and **4**, which have a methyl group on different locations (α, β, and γ, respectively, from the surface-bound S, Figure 1). To investigate the effects of surface ligand structure independent to nanoparticle size and ligand density, we have prepared a series of PdNPs with similar core sizes and surface ligand density of the constitutional isomers of pentanethiolate. The isolation of the effects of ligand-substrate steric interference from those of electronic effects related to nanoparticle size and ligand density has not been reported on soluble metal nanoparticle catalytic systems and is needed to truly understand the influence of ligand structure on nanoparticle catalysis. The spatial control over the ligand structure of nanoparticles would be especially beneficial for understanding how to control the nano-environment of reaction sites. The results show that the surrounding ligands of PdNP catalysts control the accessibility of incoming substrates to the reactive surface sites.

## Experimental

### Materials

The following materials were purchased from manufacturers and used as is: 1-Bromopentane, 2-bromopentane, tetraoctylammonium bromide (TOAB), 2-methyl-1-pentene, 3,3-dimethyl-1-butene, and sodium thiosulfate pentahydrate were obtained from Sigma-Aldrich. 1-Bromo-2,2-dimethylpropane, 1-bromo-3-methylbutane, potassium tetrachloropalladate (K_2_PdCl_4_), 1-pentene, and 3-methyl-1-pentene were obtained from Acros. 1-Bromo-2-methylbutane was purchased from Alfa Aesar. Sodium borohydride (NaBH_4_) was obtained from Fisher Scientific. Chloroform-d and deuterium oxide were purchased from Chembridge Isotope Laboratories. Water was purified using a Barnstead NANOpure diamond water system.

### Synthesis of Pentyl Thiosulfate Ligand Isomers

A 20 mmol of bromoalkane in 40 mL ethanol and 20 mmol (4.964 g) of Na_2_S_2_O_3_ in 40 mL nanopure water were added to a 500 mL round bottom flask equipped with a reflux condenser. The mixture was refluxed for 3 h and then solvent was removed under vacuum. The crude product was dissolved in hot ethanol and left to recrystallize overnight at room temperature and then in an ice bath. The product was obtained by vacuum filtration. The filtrate was dissolved in hot ethanol again for a second recrystallization. The recrystallized products were placed under 25 psi of vacuum to dry before use. ^1^H NMR spectra were collected for each ligand (Figure S1).

### Synthesis of Pd Nanoparticles

K_2_PdCl_4_ (0.4 mmol) in 12 mL nanopure water was added to 25 mL of TOAB (2.0 mmol) in 25 mL toluene in a 50 mL round bottom flask. The reaction mixture was stirred for 10 min and the aqueous layer discarded. Sodium S-pentyl thiosulfate ligand (or an isomer) (1.2 mmol) dissolved in 10 mL of 25% methanol was added to the reaction flask. Additional TOAB (2.0 mmol) was added and the reaction mixture was stirred for 15 min after the addition of NaBH_4_ (4.0 mmol) dissolved in 5 mL water. The aqueous layer was discarded again and the organic solvents removed under vacuum. The crude product was dissolved in dichloromethane and filtered through a fine frit funnel. The filtrate was collected and solvent removed under vacuum. The crude product was transferred to a centrifuge with 10 mL methanol and underwent a washing process consisting of centrifugation for 20 min, decantation of the supernatant, and suspension via 10 min of sonication. The crude product was washed with the previous method twice with methanol and twice with ethanol. The purified PdNPs were placed under 25 psi of vacuum to dry. ^1^H NMR spectra were collected for each PdNP (Figure S2). The ^1^H NMR spectra of PdNPs displayed two small peaks at ~0.8 ppm for methyl and ~1.3 ppm for methylenes (and another peak at ~1.6 ppm for PdNP capped with 1-pentanethiolate). The broadening/absence of signals corresponding to α- and β-CH_n_ from S indicated that the hydrocarbon species derived from alkyl thiosulfates are attached to the nanoparticle.

### Characterization of Pentyl Thiosulfate Ligands and Pd Nanoparticles

Proton NMR spectra were acquired on a Bruker AC400 spectrometer at 400 MHz in deuterium oxide for the ligands and chloroform-d for the nanoparticles. Thermogravimetric analysis (TGA) data was obtained from a TA Instruments SDT Q600 with a flow rate of 100 mL/min of N_2_ and heating from room temperature to 900°C. Transmission electron microscopy (TEM) images were obtained using JEOL 1200 EX II electron microscope operating at 80 kV.

### Catalytic Isomerization/Hydrogenation Reactions

A 3 mL of CDCl_3_ with 5 mol % PdNPs was placed in a small round bottom flask with a rubber septum. The flask was purged with 1 atm H_2_ for 10 min. The alkene substrate (0.40 mmol) was injected into the flask after the purge. The reaction was continuously stirred at room temperature. Aliquots of the reaction were removed at 0, 1, 3, 6, and 24 h after the start of the reaction to obtain proton NMR spectra. A brief second H_2_ purge was performed after the 6 h reaction.

## Results and Discussion

### Synthesis and Characterization of Pd Nanoparticles Capped With Constitutional Isomers of Pentanethiolate Ligands

**Figure 2:**
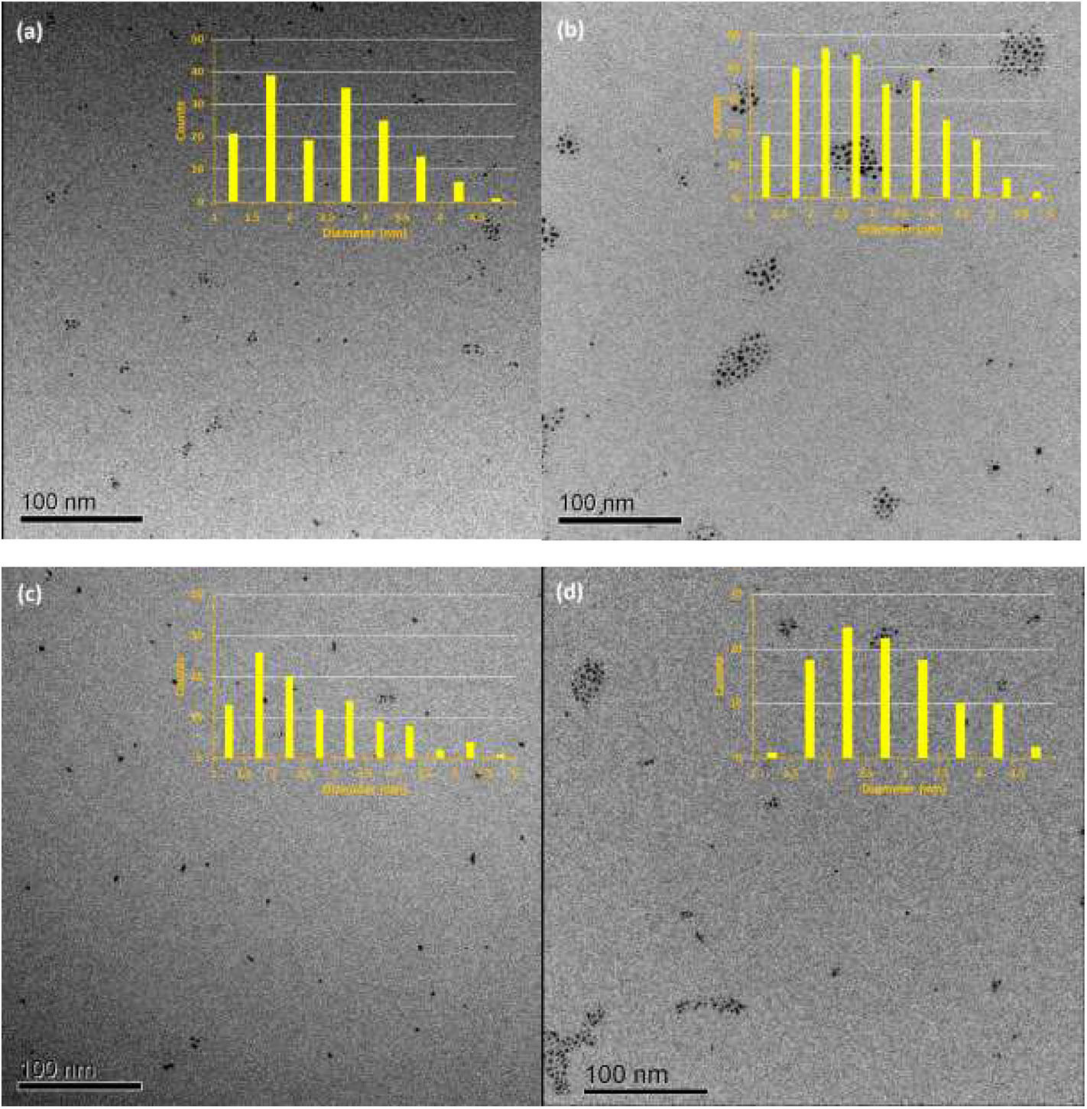
TEM images and histograms of four synthesized palladium nanoparticles: **(a)** PdNP, **(b)** PdNP_α_, **(c)** PdNP_β_, and **(d)** PdNP_γ_.

PdNPs capped with constitutional isomers of pentanethiolate ligands were synthesized using the published procedure from our lab with a modified mole ratio of ${\text{PdCl}}_{4}^{2-}$ to surfactants (TOAB and alkylthiosulfate) (Maung and Shon, 2017). The two-phase method using two equivalents of sodium S-pentyl thiosulfate isomers to palladium metal complex initially yielded nanoparticles with less desirable stability and solubility in organic solvents. The ligands with short alkyl chain (5 or less) didn't provide necessary passivation efficiency at the specified concentration, requiring the higher ratio of alkyl thiosulfate precursor ligands. Overall, the optimal condition for synthesis of pentanethiolate-capped PdNPs was with 10 molar equivalents of TOAB and 3 molar equivalents of alkyl thiosulfate. This condition was also used for the synthesis of other PdNPs using constitutional isomers of pentyl thiosulfate. In addition to achieving the necessary stability in organic solvents, the surface ligand density should be similar between the nanoparticles to minimize variability on catalytic activity due to the surface density (Vargas et al., 2019). Since the constitutional isomers of ligands are used for surface capping, the direct comparison of organic weight fraction to metal fraction would be sufficient as long as the average core sizes of nanoparticles are comparable. To this end, a combination of TEM and TGA was used to confirm the similarities in the size and ligand content of PdNPs capped with pantanethiolate isomers (Table 1). TEM images and TGA results of each PdNP are shown in Figure 2 (additional TEM images in Figure S3) and Figure S4, respectively. The characterization of these PdNPs showed that they have similar average core sizes (2.6–2.9 nm) and organic ligand contents (21–25% pentanethiolate isomers in weight fraction). TEM images showed that all nanoparticles are spherical and well-dispersed, but the formation of small domains of nanoparticles were more frequently observed for PdNPs capped with 1-methylbutanethiolate (PdNP_α_). The histogram analyses obtained from multiple TEM images for each PdNP indicated that the size dispersity of nanoparticles within each sample (as quantified by standard deviation) is relatively large. This is due to the poor initial passivation capability of small alkyl thiosulfate ligands during nucleation-growth of Pd nanoparticles.

**Table 1:** % Pd and average core size of four synthesized palladium nanoparticles.

**Surface structure**	**PdNP label**	**% Pd**	**Core size (diameter, nm)**
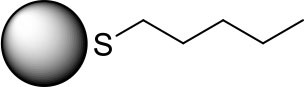	PdNP	79	2.6 ± 0.9
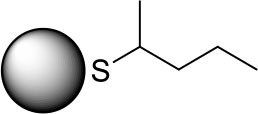	PdNP_α_	79	2.9 ± 1.1
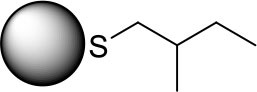	PdNP_β_	75	2.7 ± 1.1
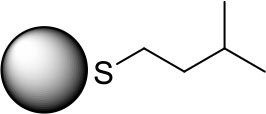	PdNP_γ_	75	2.8 ± 0.8

The overall colloidal stability of PdNPs with different pentanethiolate constitutional isomers was observed in solution phase. Visually, the PdNPs capped with pentanethiolate (PdNP), 2-methylbutanethiolate (PdNP_β_), and 3-methylbutanethiolate (PdNP_γ_) remain soluble in organic solvents, such as chloroform for a long term and maintain colloidal stability even after catalytic reaction. However, PdNPs capped with 1-methylbutanethiolate (PdNP_α_) frequently lost their colloidal stability during the catalytic reactions and formed precipitates, indicating possible particle agglomeration through core interactions. The characterization of PdNP_α_ by TEM analysis (Figure S5) performed after the catalytic reaction indicated the aggregation of PdNP. Due to the separation of nanoparticles from the reaction solution, the UV-vis spectrum of the solution after the catalytic reaction did not show any spectral feature corresponding to PdNP_α_. These are clear indications that the PdNP_α_ undergo irreversible aggregation and loose its homogeneous characteristics. The difference in colloidal stability of PdNPs indicated that the surface coordination of secondary alkanethiolate is more fragile than that of primary alkanethiolate ligands due to the smaller van der Waals interactions between sterically bulky and small secondary alkanethiolate ligands.

### Catalysis of Pd Nanoparticles

**Scheme 1:**
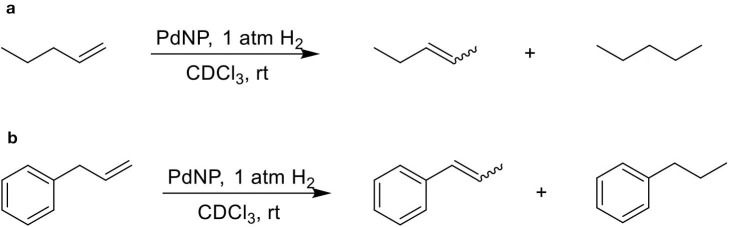
The model catalytic reactions used to compare the activity and selectivity of colloidal Pd nanoparticle catalysts: **(a)** pen-1-ene and **(b)** allylbenzene.

The isomerization/hydrogenation of pent-1-ene can be used to probe the influence of surface ligand structure on activity in addition to selectivity due to the potential for two different organic transformations. It has been found that octanethiolate-capped PdNPs could convert 99% of pent-1-ene to either the isomerization or hydrogenation product in 24 h at room temperature and under atmospheric H_2_ pressure (Zhu and Shon, 2015). High selectivity toward the isomerization product (91%) compared to the hydrogenation product was observed in this previous study. The reaction of pent-1-ene (Scheme 1a) was investigated by each of the four PdNPs capped with constitutional isomers of pentanethiolate and the catalytic data were acquired after 24 h. Due to the poor colloidal stability of PdNP_α_, the catalytic reactions using PdNP_α_ produced inconsistent and generally poor results ranging from no conversion to yields close to those of PdNP_β_. For systematic comparison, our discussion focuses on more stable PdNPs (PdNP, PdNP_β_, and PdNP_γ_) that maintain homogeneous condition and the results are shown in Table 2.

**Table 2:** The catalysis results for pentene and allylbenzene derivatives by colloidal Pd nanoparticles^a^.

**Substrates**	**Products**	**C5 PdNP**	**C5_**β**_ PdNP**	**C5_**γ**_ PdNP**
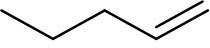	Conversion	100	22	100
**1**	Isomerization	97	21	89
	Hydrogenation	3	1	11
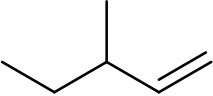	Conversion	86	33	94
	Isomerization	48	17	48
**2**	Hydrogenation	38	16	46
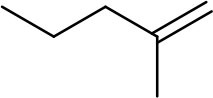	Conversion	59	8	52
**3**	Isomerization	59	8	52
	Hydrogenation	0	0	0
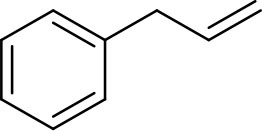	Conversion	100	89	100
	Isomerization (trans + cis)	88 (85 + 3)	84 (70 + 14)	92 (86 + 6)
**4**	Hydrogenation	12	5	8
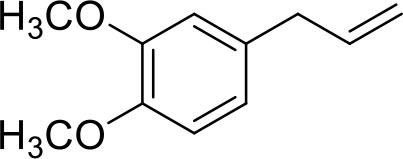	Conversion	100	70	91
	Isomerization (trans + cis)	80 (76 + 4)	63 (51 + 12)	83 (72 + 11)
**5**	Hydrogenation	20	7	8

a *Reaction condition: 5 mol% PdNPs, room temperature, CDCl_3_ solvent, and 1 atm H_2_ for 24 h. The yields are obtained via ^1^H NMR spectra analysis (Figures S6, S7)*.

**Scheme 2:**
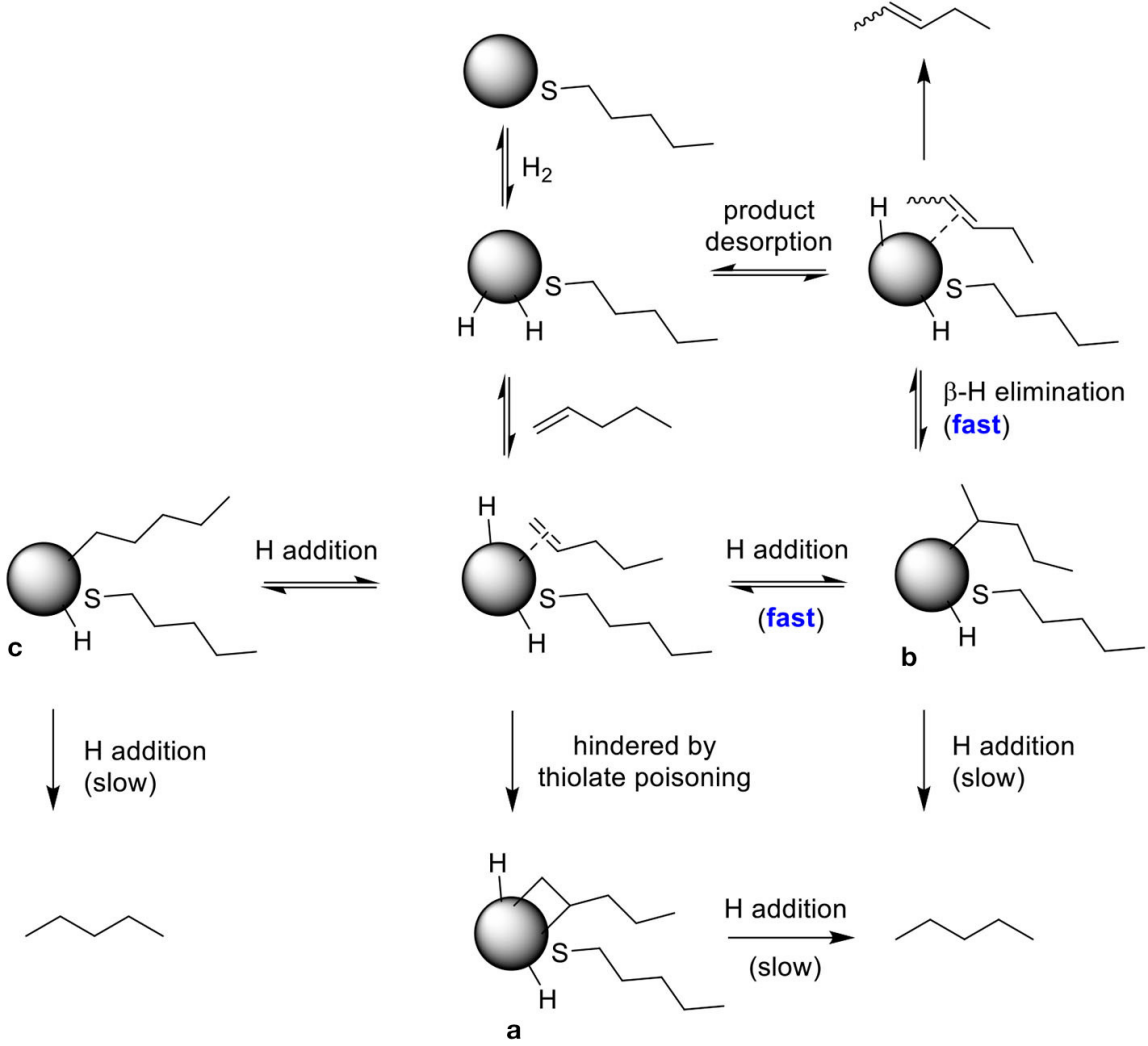
The proposed reaction pathways describing the selectivity of PdNP for isomerization of pent-1-ene.

The competition between hydrogenation and isomerization of pent-1-ene is a regioselective process with the mechanism involving different palladium-alkyl intermediates as shown in Scheme 2. The higher selectivity for isomerization reported in Table 2 for all PdNPs is due to the surface crowding and partial poisoning effects by alkanethiolate ligands, which limit the formation of di-σ-bonded intermediate **a** and kinetically hinder the simultaneous addition of two H. The addition of single H can result in the formation of either branched mono σ-bonded Pd alkyl intermediate **b** or linear mono σ-bonded Pd alkyl intermediate **c**. The formation of branched σ-bonded Pd alkyl intermediate is kinetically more favorable because H is added to the less substituted carbon. The surface crowding effect also hinders the addition of second H to the Pd alkyl intermediate making this process slower than β-H elimination reaction.

**Figure 3:**
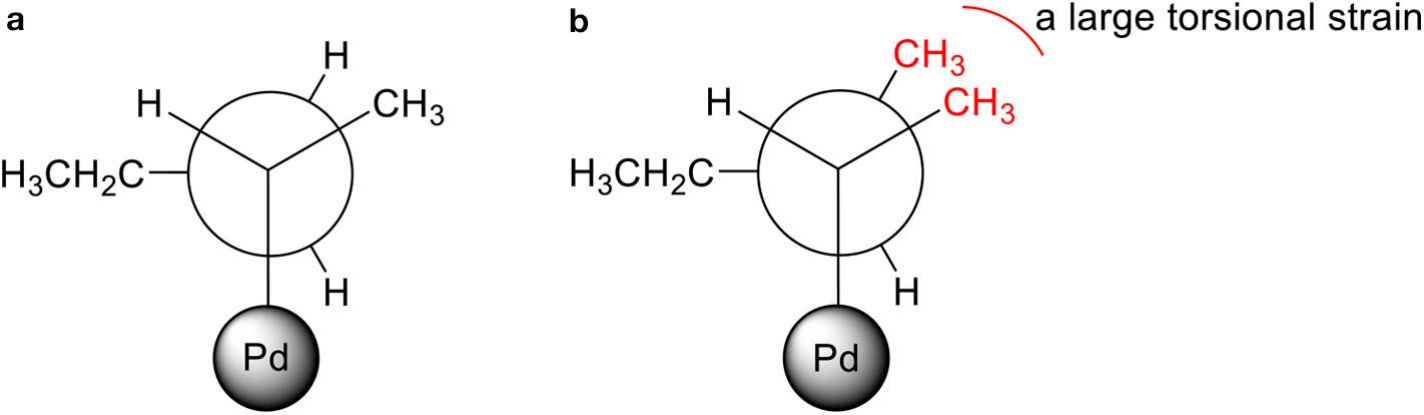
Newman projections of branched mono-σ bonded Pd-alkyl intermediates in totally eclipsed confirmations: **(a)** pent-1-ene and **(b)** 3-methylpent-1-ene.

For PdNP and PdNP_γ_, pent-1-ene (**1**) was fully converted to the isomerization or hydrogenation products. In contrast, the catalytic reaction of PdNP_β_ resulted in only ~22% conversion of pent-1-ene indicating the presence of large steric interactions between the small substrate and the bulkier surface ligand, 2-methylbutanethiolate. The steric interference to the formation of the Pd-alkyl intermediate by bulky substrates, such as 3,3-diemthylbut-1-ene and stilbene has previously been observed in the work done by (Zhu and Shon, 2015). The results shown in this work also indicated that the presence of methyl group on γ position from thiolate group has only subtle effect on the activity and selectivity between isomerization and hydrogenation during the catalytic reaction of pent-1-ene. When 3-methylpent-1-ene (**2**) was used as a substrate, the catalytic reactions of PdNP and PdNP_γ_ also resulted in better conversion yields (86 and 94%, respectively) than that of PdNP_β_ (33%). The large decrease in the selectivity for isomerization of 3-methylpent-1-ene (~48%) to that of pent-1-ene (~97%) was due to the presence of single α hydrogen in 3-methylpent-1-ene compared to two α hydrogen in pent-1-ene. This should decrease the rate for β-H elimination process and provide an opportunity for second H addition to surface coordinated carbon of the mono-σ bonded intermediate based on the mechanism shown in Scheme 2. The more branched 3-methylpent-1-ene substrate would also have a higher energy barrier to orientate in the eclipsed manner before undergoing β-H elimination as shown in Figure 3. In addition, the steric interaction of branched mono-σ bonded intermediate with surface ligand would be much greater for 3-methylpent-1-ene compared to pent-1-ene. The catalysis of 2-methylpent-1-ene (**3**), which is a tri-substituted alkene, was much slower than those of the other two pent-1-ene substrates. After 24 h the total conversions of 2-methylpent-1-ene were limited to 59, 8, and 52% for PdNP, PdNP_β_, and PdNP_γ_, respectively. The same trend of lower activity was observed for the catalytic reaction of PdNP_β_ confirming the steric interference of 2-methylbutanethiolate ligand. Another interesting result for the catalytic reactions of 2-methylpent-1-ene was the absence of any hydrogenation product for all three catalysts. This result suggested that the formation of branched mono-σ bonded intermediate is much more favorable than the formation of either di-σ-bonded intermediate or linear mono-σ-bonded intermediate. This indicated the H addition to tri-substituted alkene carbon is kinetically much slower than the H addition to terminal alkene carbon.

The catalytic reaction of allylbenezene (**4**) was also used as a model system to investigate the influence of steric methyl branch group of PdNPs with different isomeric thiolate ligands (Scheme 1b). Our previous studies have shown that the catalytic isomerization of allylbenzene was found to be a major reaction pathway for octanethiolate-capped PdNPs (Zhu and Shon, 2015). For both PdNP and PdNP_γ_, the catalytic conversion of allylbenzene was completed in 24 h (100% to either isomerization or hydrogenation products). The similar selectivity for isomerization was also observed for PdNP and PdNP_γ_ (88 and 92%, respectively), with the thermdynamic *trans* isomers being the major products, over hydrogenation (12 and 8%, respectively). In contrast, the catalytic reaction of PdNP_β_ resulted in an incomplete conversion (89%). The higher selectivity for isomerization (84%) over hydrogenation (5%), however, was remained same for PdNP_β_ compared to PdNP and PdNP_γ_. The increased activity of PdNP_β_ for allylbenzene compared to other three aliphatic alkenes discussed above indicated that the presence of phenyl group in allylbenzene enhance the activity of PdNP_β_ due to the stronger substrate adsorption of allylbenzene on the surface of Pd nanoparticles. The increased hydrogenation activity of aromatic alkenes over aliphatic alkenes for metal catalysts has been previously reported (Zhu and Shon, 2015). The catalytic reactions of 1-allyl-3,4-dimethoxybenzene (**5**) produced similar results with slightly decreased activity and selectivity. For PdNP and PdNP_γ_, the catalytic reactions resulted in 100 and 92% conversions, respectively, with the isomerization being the major products (80 and 83%, respectively). PdNP_β_ was less active converting only 69% of 1-allyl-3,4-dimethoxybenzene to either the isomerization (63%) and the hydrogenation (7%) products. We propose that the slightly decreased activity observed for the catalytic reaction of 1-allyl-3,4-dimethoxybenzene compared to that of allylbenzene is due to the steric interference of dimethoxy groups with surface ligands slightly hindering the surface adsorption of phenyl group on palladium nanoparticles. This steric interference, therefore, reduces the positive effect of phenyl group observed from the catalytic reactions of allylbenzene.

## Conclusions

In summary, four different nanoparticles capped with the constitutional isomers of pentanethiolate were successfully synthesized. Among these palladium nanoparticles capped with secondary pentanethiolate exhibited a poor colloidal stability often precipitating out during the catalytic reactions. Three stable Pd nanoparticles capped with primary pentanethiolate isomers were used to investigate the influence of steric hindrance near the metal surface. Generally, Pd nanoparticles with thiolate ligands with a methyl substituent at the gamma location had no influence on the activity and selectivity comparable to Pd nanoparticles with the straight chain. However, the presence of thiolate ligands with a methyl substituent at the beta position decreased the activity of Pd nanoparticles greatly. The activity and selectivity of Pd nanoparticles were also greatly affected by the structure and size of various terminal alkenes. This study clearly indicates that the interaction between the surface ligands and substrates is important in determining the overall activity of nanoparticle catalysts. Further systematic investigations on the ligand structure-catalysis function relationships will be necessary for advancing colloidal metal nanoparticles as industrially applicable high regioselective, chemoselective, and/or stereoselective catalytic systems.

## Data Availability Statement

The raw data supporting the conclusions of this article will be made available by the authors, without undue reservation.

## Author Contributions

PT and VN conducted the experimental work. PT wrote the original draft and developed the experimental methodology. Y-SS supervised the project, provided the resources, and edited the draft. All authors contributed to the article and approved the submitted version.

## Conflict of Interest

The authors declare that the research was conducted in the absence of any commercial or financial relationships that could be construed as a potential conflict of interest.

## References

[B1] Campisi S. Schiavoni M. Chan-Thaw C. E. Villa A. (2016). Untangling the role of the capping agent in nanocatalysis: recent advances and perspectives. Catalysts 6:185 10.3390/catal6120185

[B2] Chen T.-A. Shon Y.-S. (2018). Alkanethiolate-capped palladium nanoparticles for regio- and stereoselective hydrogenation of allenes. Catalysts 8:428. 10.3390/catal8100428 30733870 PMC6363366

[B3] Chen V. Pan H. Jacobs R. Derakhshan S. Shon Y.-S. (2017). Influence of graphene oxide supports on solution-phase catalysis of thiolate-protected palladium nanoparticles in water. New J. Chem. 41, 177–183. 10.1039/C6NJ02898E 28652688 PMC5482534

[B4] Du Y. Sheng H. Astruc D. Zhu M. (2020). Atomically precise noble metal nanoclusters as efficient catalysts: a bridge between structure and properties. Chem. Rev. 120, 526–622. 10.1021/acs.chemrev.8b00726 30901198

[B5] Elliott E. W. III. Glover R. D. Hutchison J. E. (2015). Removal of thiol ligands from surface-confined nanoparticles without particle growth or desorption. ACS Nano 9, 3050–3059. 10.1021/nn5072528 25727562

[B6] Gauthier D. Lindhardt A. T. Olsen E. P. K. Overgaard J. Skrydstrup T. (2010). *In situ* generated bulky palladium hydride complexes as catalysts for the efficient isomerization of olefins. Selective transformation of termainal alkenes to 2-alkenes. J. Am. Chem. Soc. 132, 7998–8009. 10.1021/ja9108424 20481527

[B7] Gavia D. J. Do Y. Gu J. Shon Y.-S. (2014). Mechanistic insights into the formation of dodecnethiolate-stabilized magnetic iridium nanoparticles: thiosulfate vs thiol ligands. J. Phys. Chem. C 118, 14548–14554. 10.1021/jp504239x 25018790 PMC4084834

[B8] Geukens I. de Vos D. E. (2013). Organic transformations on metal nanoparticles: controlling activity, stability, and recyclability by support and solvent interactions. Langmuir 29, 3170–3178. 10.1021/la304639z 23331049

[B9] Goodman E. D. Schwalbe J. A. Cargnello M. (2017). Mechanistic understanding and the rational design of sinter-resistant heterogeneous catalysts. ACS Catal. 7, 7156–7173. 10.1021/acscatal.7b01975

[B10] Heiligtag F. J. Niederberger M. (2013). The fascinating world of nanoparticle research. Mater. Today 16, 262–271. 10.1016/j.mattod.2013.07.004

[B11] Henkel C. Wittmann J. E. Träg J. Will J. Stiegler L. M. S. Strohriegl P.. (2020). Mixed organic ligand shells: controlling the nanoparticle surface morphology toward tuning the optoelectronic properties. Small 16, 1903729. 10.1002/smll.201903729 31778297

[B12] Heuer-Jungemann A. Feliu N. Bakaimi I. Hamaly M. Alkilany A. Chakraborty I.. (2019). The role of ligands in the chemical synthesis and applications of inorganic nanoparticles. Chem. Rev. 119, 4819–4880. 10.1021/acs.chemrev.8b00733 30920815

[B13] Ingham B. Lim T. H. Dotzler C. J. Henning A. Toney M. F. Tilley R. D. (2011). How nanoparticles coalesce: an *in situ* study of Au nanoparticle aggregation and grain growth. Chem. Mater. 23, 3312–3317. 10.1021/cm200354d

[B14] King S. R. Gentle A. R. Gortie M. B. McDonagh A. M. (2018). On the development of optical properties during thermal coarsening of gold nanoparticle composites. J. Phys. Chem. C 122, 12098–12105. 10.1021/acs.jpcc.8b02744

[B15] Kister T. Monego D. Mulvaney P. Widmer-Cooper A. Kraus T. (2018). Colloidal stability of apolar nanoparticles: the role of particle size and ligand shell structure. ACS Nano 12, 5969–5977. 10.1021/acsnano.8b02202 29842786

[B16] Lohse S. E. Murphy C. J. (2012). Applications of colloidal nanoparticles: from medicine to energy. J. Am. Chem. Soc. 134, 15607–15620. 10.1021/ja307589n 22934680

[B17] Lu L. Lou B. Zou S. Kobayashi H. Liu J. Xiao L. (2018). Robust removal of ligands from noble metal nanoparticles by electrochemical strategies. ACS Catal. 8, 8484–8492. 10.1021/acscatal.8b01627

[B18] Mahdaly M. A. Zhu J. S. Nguyen V. Shon Y.-S. (2019). Colloidal palladium nanoparticles for selective hydrogenation of styrene derivatives with reactive functional groups. ACS Omega 4, 20819–20828. 10.1021/acsomega.9b03335 31858068 PMC6906945

[B19] Maung M. S. Dinh T. Salazar C. Shon Y.-S. (2017). Unsupported micellar palladium nanoparticles for biphasic hydrogenation and isomerization of hydrophobic allylic alcohols in water. Coll. Surf. A 513, 367–372. 10.1016/j.colsurfa.2016.10.067 28579696 PMC5450820

[B20] Maung M. S. Shon Y.-S. (2017). Effects of noncovalent interactions on the catalytic activity of unsupported colloidal palladium nanoparticles stabilized with thiolate ligands. J. Phys. Chem. C 121, 20882–20891. 10.1021/acs.jpcc.7b07109 29326755 PMC5758047

[B21] Pang S. H. Lien C.-H. Medlin J. W. (2016). Control of surface alkyl catalysis with thiolate monolayers. Catal. Sci. Technol. 6, 2413–2418. 10.1039/C5CY01831E25353667

[B22] Pensa E. Cortés E. Corthey G. Carro P. Vericat C. Fonticelli M. H.. (2012). The chemistry of the sulfur-gold interface: in search of a unified model. Acc. Chem. Res. 45, 1183–1192. 10.1021/ar200260p 22444437

[B23] Rossi L. M. Fiorio J. L. Garcia M. A. S. Ferraz C. P. (2018). The role and fate of capping ligands in colloidally prepared metal nanoparticle catalysts. Dalton Trans. 47, 5889–5915. 10.1039/C7DT04728B 29509204

[B24] San K. A. Chen V. Shon Y.-S. (2017). Preparation of partially poisoned alkanethiolate-capped platinum nanoparticles for hydrogenation of activated terminal alkynes. ACS Appl. Mater. Interfaces 9, 9823–9832. 10.1021/acsami.7b02765 28252941 PMC5364944

[B25] San K. A. Shon Y.-S. (2018). Synthesis of alkanethiolate-capped metal nanoparticles using alkyl thiosulfate ligand precursors: a method to generate promising reagents for selective catalysis. Nanomaterials 8:346. 10.3390/nano8050346 29783714 PMC5977360

[B26] Shon Y.-S. Choi D. Dare J. Dinh T. (2008). Synthesis of nanoparticle-cored dendrimers by convergent dendritic functionalization of monolayer-protected nanoparticles. Langmuir 24, 6924–6931. 10.1021/la800759n 18507425

[B27] Vargas K. M. San K. A. Shon Y.-S. (2019). Isolated effects of surface ligand density on the catalytic activity and selectivity of palladium nanoparticles. ACS Appl. Nano Mater. 2, 7188–7196. 10.1021/acsanm.9b01696 PMC8171273 34085029

[B28] Zhang F. Zheng S. Xiao Q. Zhong Y. Zhu W. Lin A. (2016). Synergetic catalysis of palladium nanoparticles encaged within amine-functionalized UiO-66 in the hydrodeoxygenation of vanillin in water. Green Chem. 18, 2900–2908. 10.1039/C5GC02615F

[B29] Zhu J. S. Shon Y.-S. (2015). Mechanistic interpretation of selective catalytic hydrogenation and isomerization of alkenes and dienes by ligand deactivated Pd nanoparticles. Nanoscale 7, 17786–17790. 10.1039/C5NR05090A 26455381 PMC5758039

