# Supplementary material for: Proximity Effects of Methyl Group on Ligand Steric Interactions and Colloidal Stability of Palladium Nanoparticles
Source: Front Chem. 2020 Jul 9;8:599. doi: 10.3389/fchem.2020.00599 (PMC7381309; doi:10.3389/fchem.2020.00599)
Supplement: Supplementary file 1 [file Data_Sheet_1.PDF]

Supplementary Material

(a)

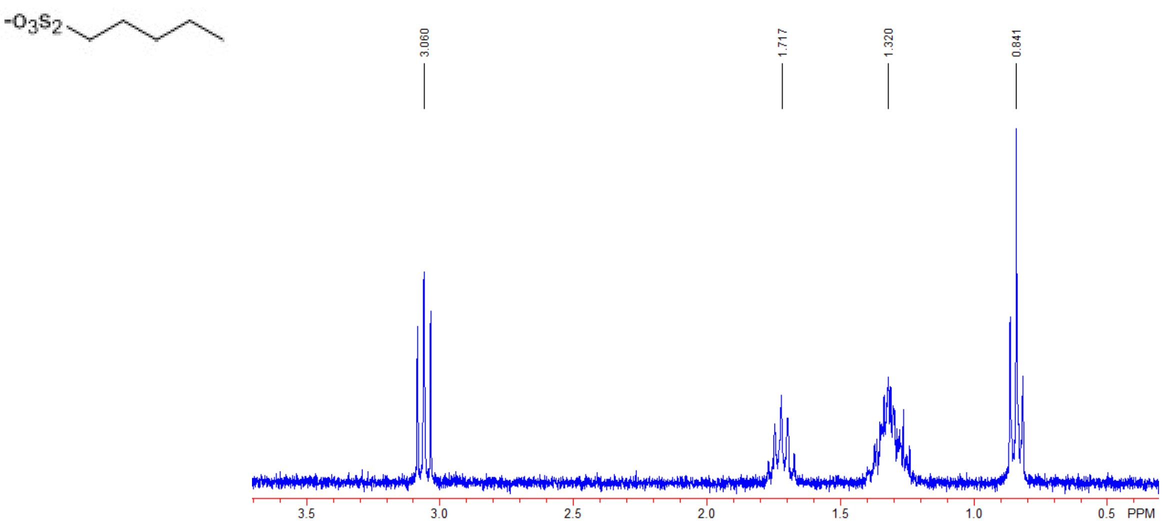

(b)

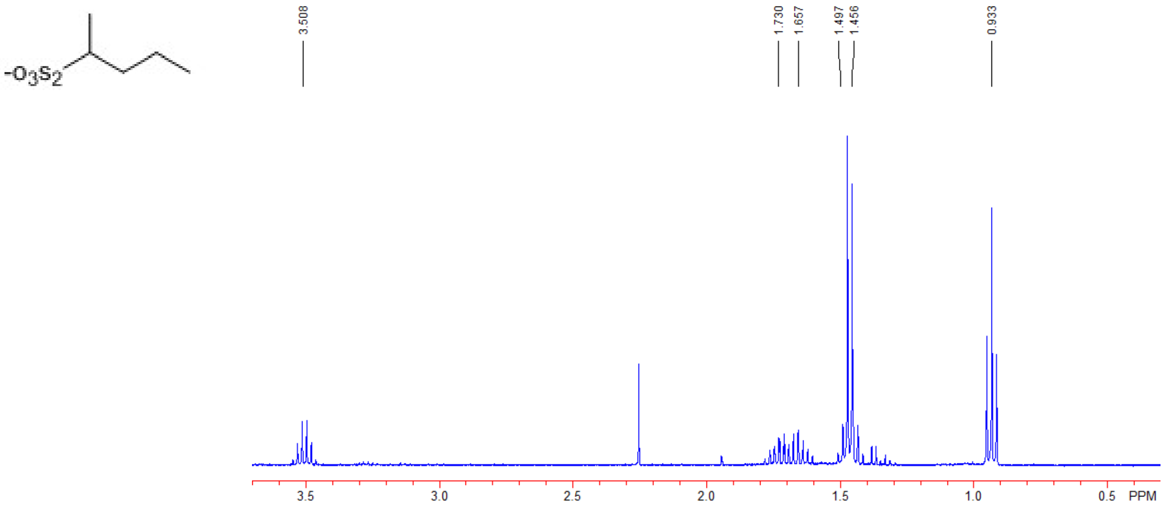

(c)

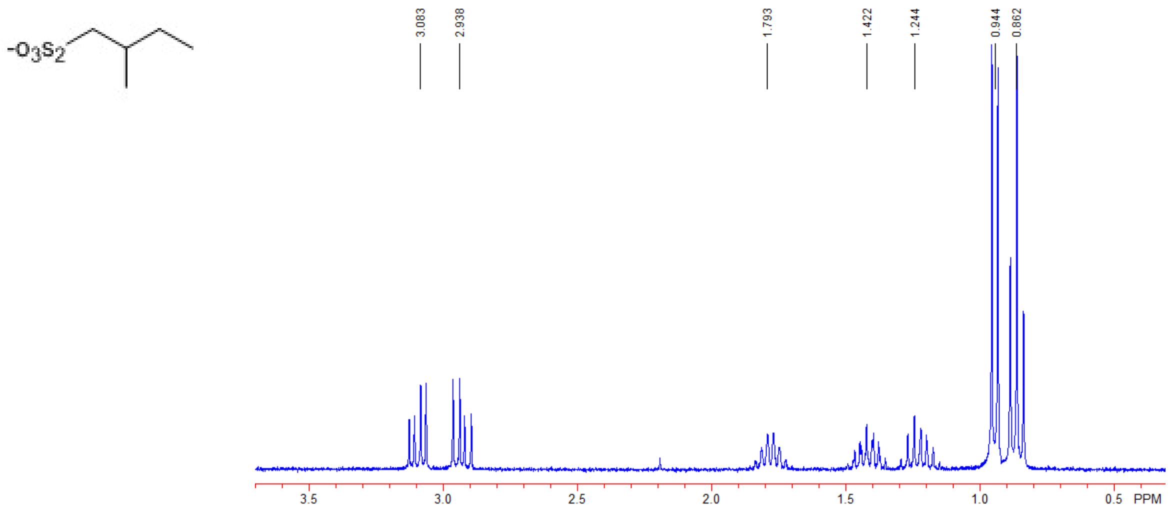

(d)

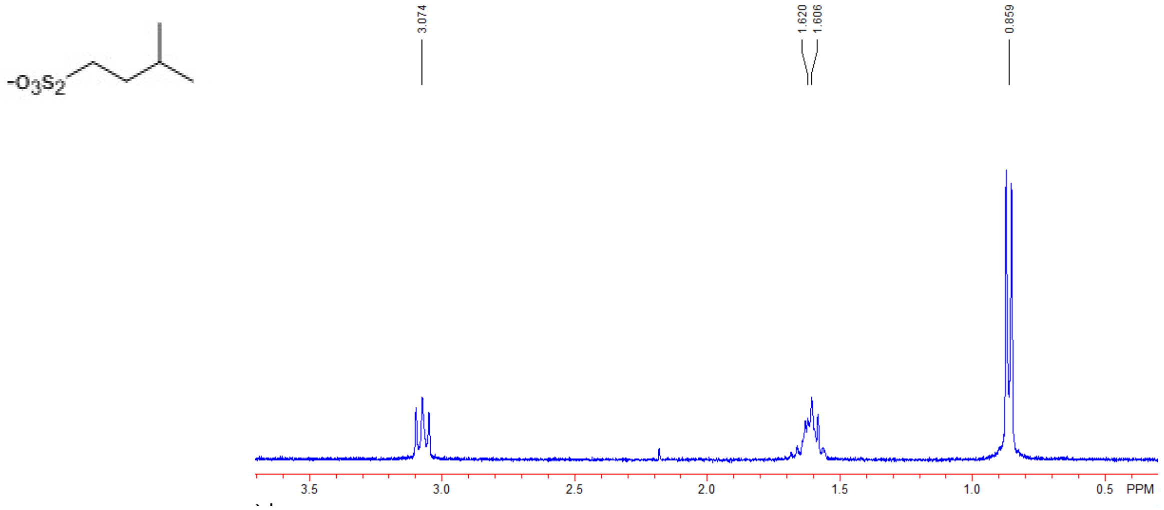

Figure S1. Proton NMR spectra of constitutional isomers of pentyl thiosulfate in D_2O: (a) Sodium S-pentyl thiosulfate, (b) sodium S-(1-methylbutyl) thiosulfate, (c) sodium S-(2-methylbutyl) thiosulfate, and (d) sodium S-(3-methylbutyl) thiosulfate.

(a)

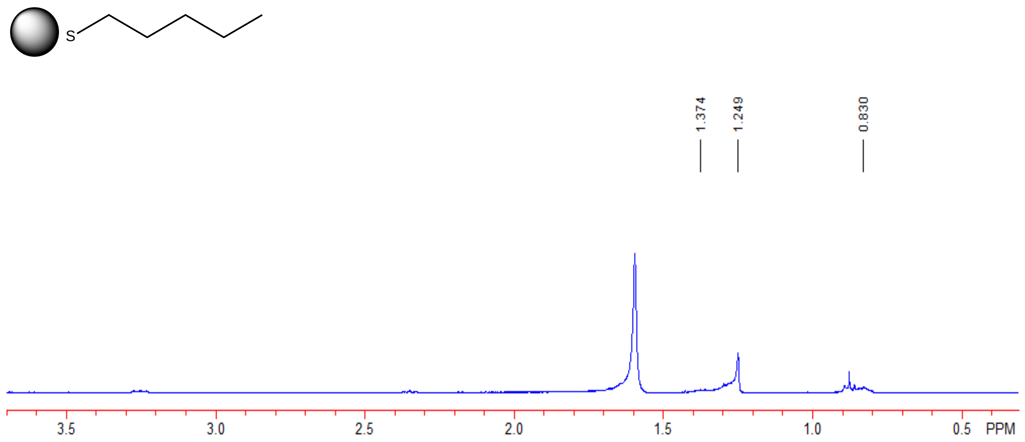
S
1.374
1.249
0.830
3.5
3.0
2.5
2.0
1.5
1.0
0.5
PPM

(b)

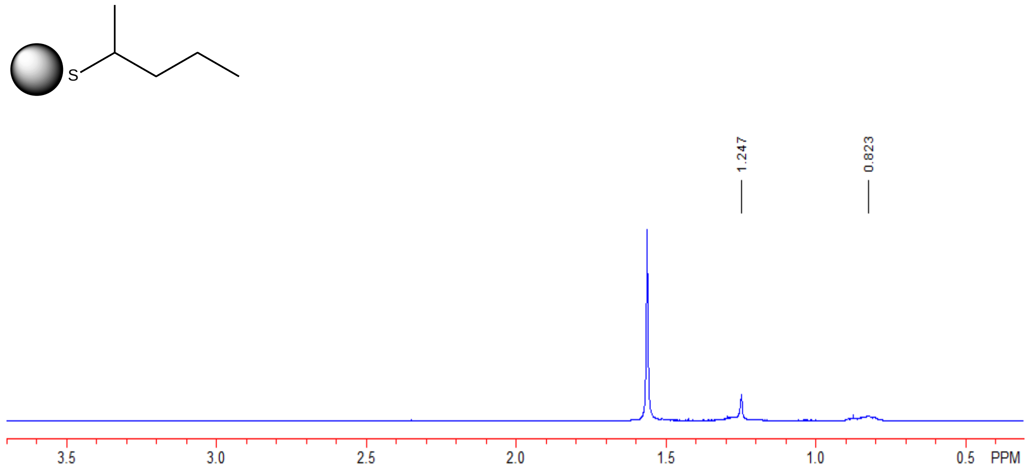
S
1.247
0.823
3.5
3.0
2.5
2.0
1.5
1.0
0.5
PPM

(c)

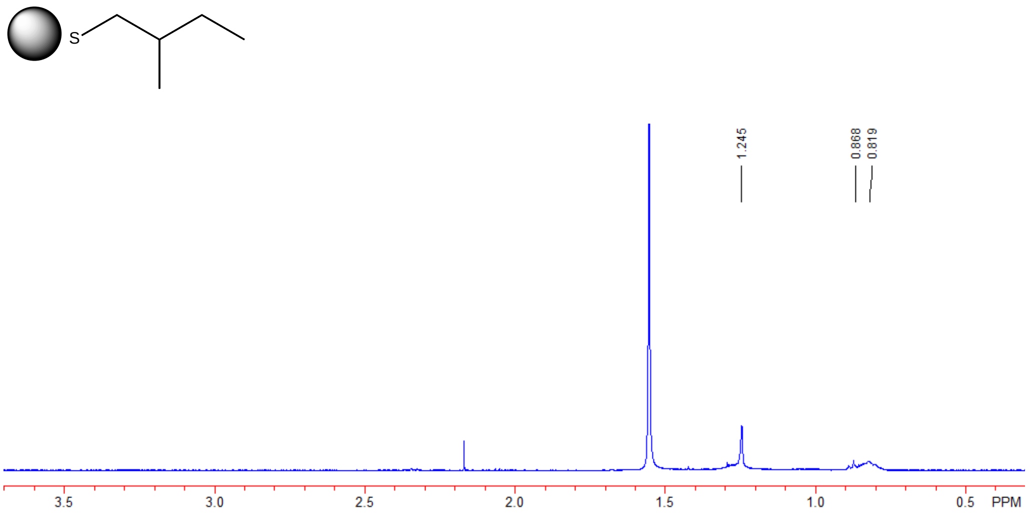

(d)

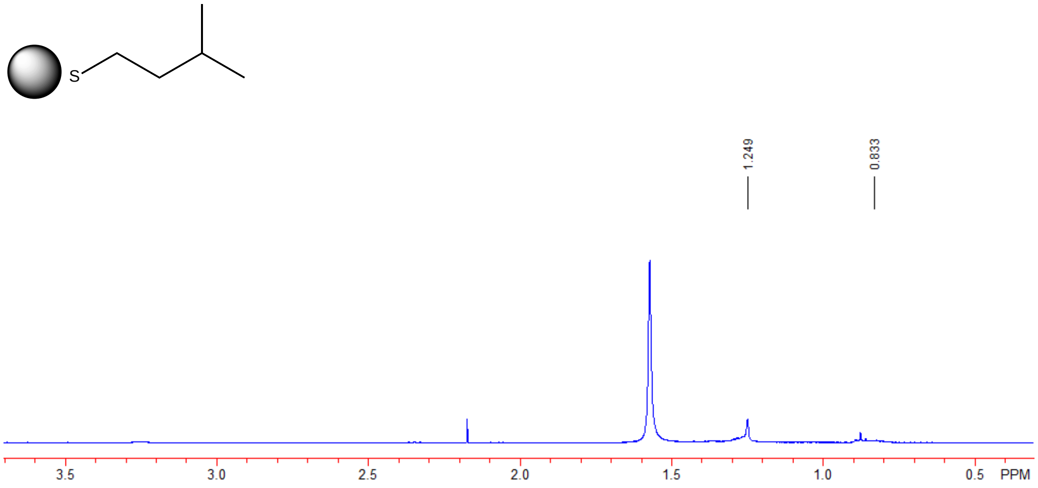

Figure S2. Proton NMR spectra of Pd nanoparticles with constitutional isomers of pentyl thiosulfate in $CDCl_3$: (a) C5 PdNP, (b) C5$_{\alpha}$ PdNP, (c) C5$_{\beta}$ PdNP, and (d) sodium S-(3-methylbutyl) thiosulfate. The α and β resonances from S are not observed from Pd nanoparticles due to the extensive broadening of signals (this is a strong evidence for ligand attachment in addition to the broadening of other signals). These characteristics make the proton NMR spectra of all PdNP almost identical. The peaks at ~1.58 ppm and 2.16 ppm are due to the presence of water and acetone, respectively. The peaks at ~3.25 ppm and the sharp peaks at ~0.88 ppm are due to the presence of residual tetraoctylammonium bromide.

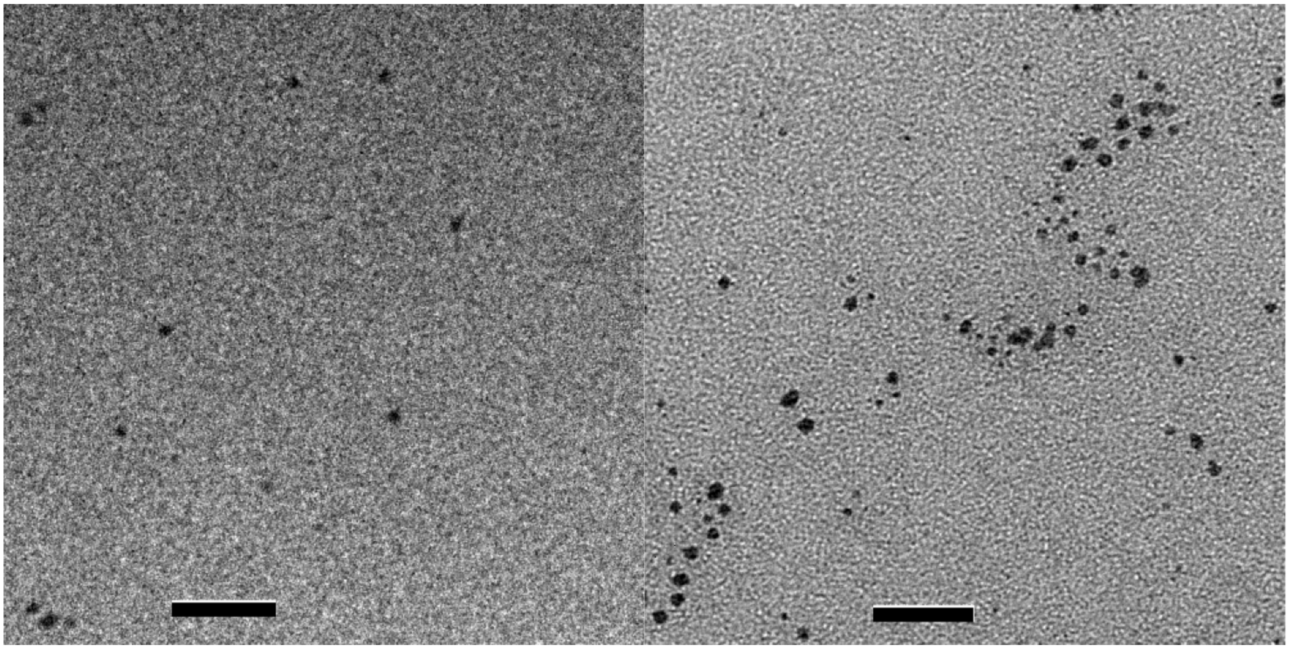

(a) (b)

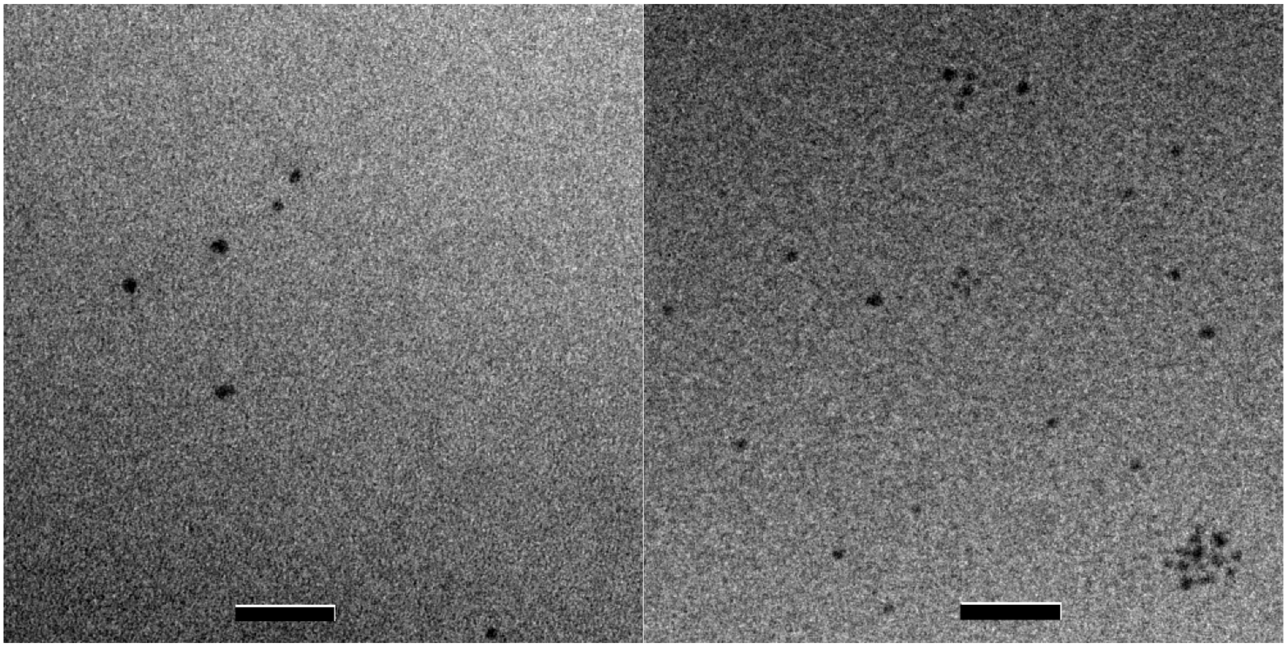

(c) (d)

Figure S3. Additional TEM images of four synthesized palladium nanoparticles: (a) PdNP (b) $PdNP_{\alpha}$, (c) $PdNP_{\beta}$, and (d) $PdNP_{\gamma}$ with higher magnification. The size bars are 25 nm.

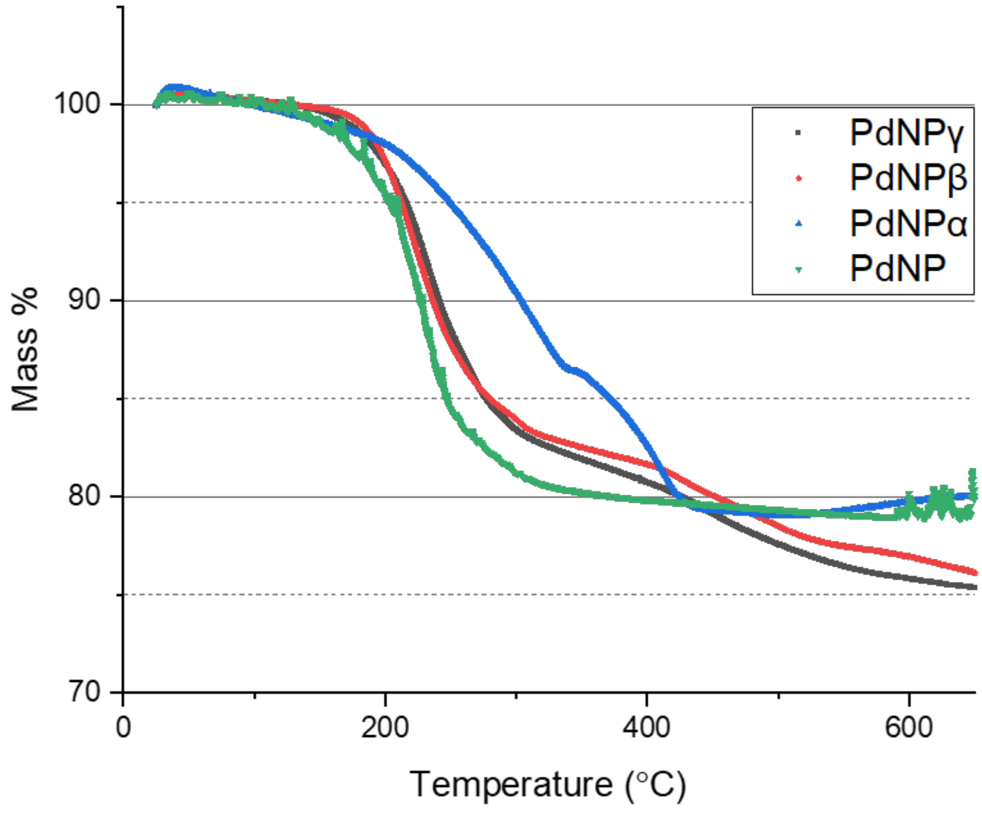

Figure S4. Thermogravimetric analysis results for PdNP, PdNP$_{\alpha}$, PdNP$_{\beta}$, and PdNP$_{\gamma}$.

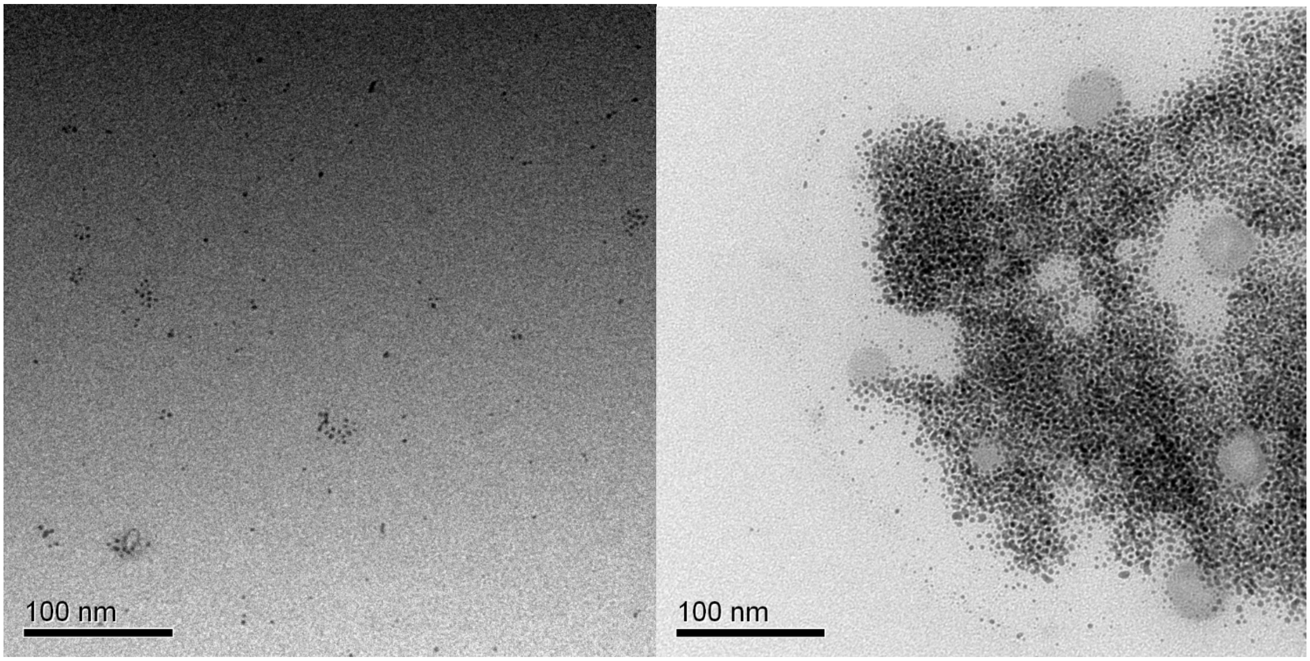

(a) (b)

Figure S5. TEM images of (a) PdNP and (b) $PdNP_{\alpha}$ after catalytic reactions showing the difference in the aggregate formation.

(a)

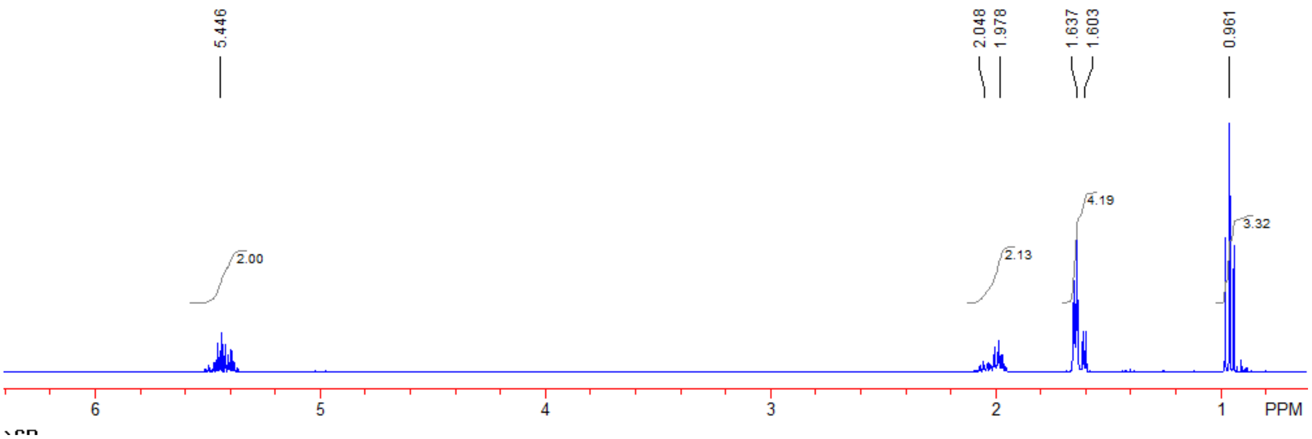

(b)

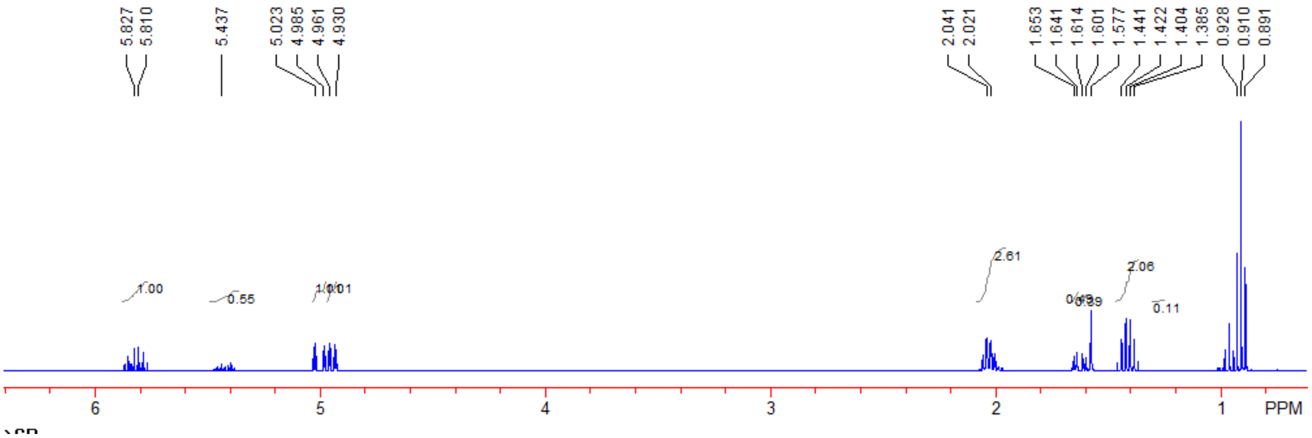

(c)

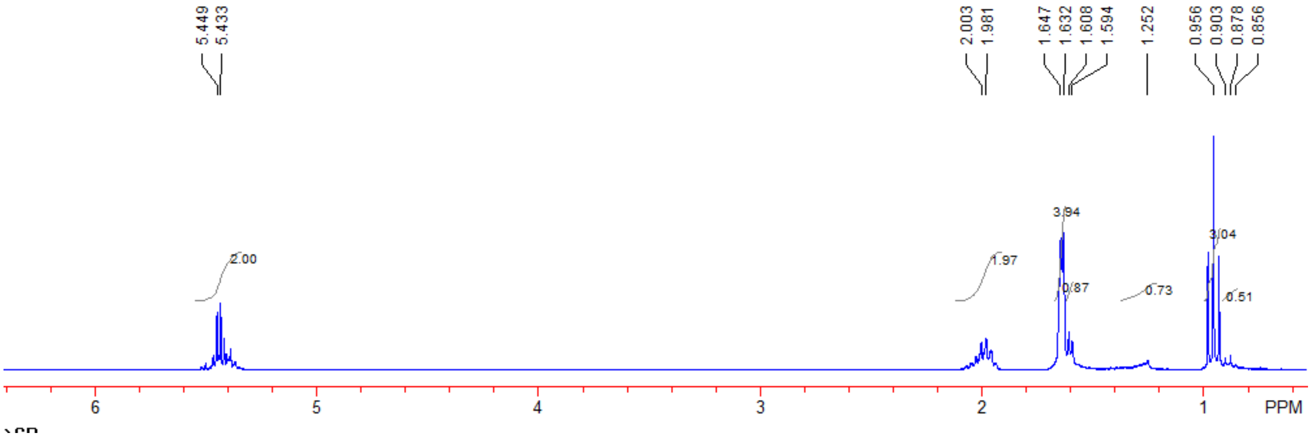

Figure S6. Proton NMR spectra obtained after reacting pent-1-ene in $CDCl_3$ under H_2 conditions after 24 h with (a) C5 PdNP, (b) C5$_\beta$ PdNP, and (c) C5$_\gamma$ PdNP. The peaks at ~5.45 ppm, 2.05 ppm, 1.98 ppm, 1.64 ppm, and 0.96 ppm are the characteristic shifts of pent-2-ene. The peaks at ~1.60 ppm and t ~0.90 ppm are the characteristic shifts of pantane.

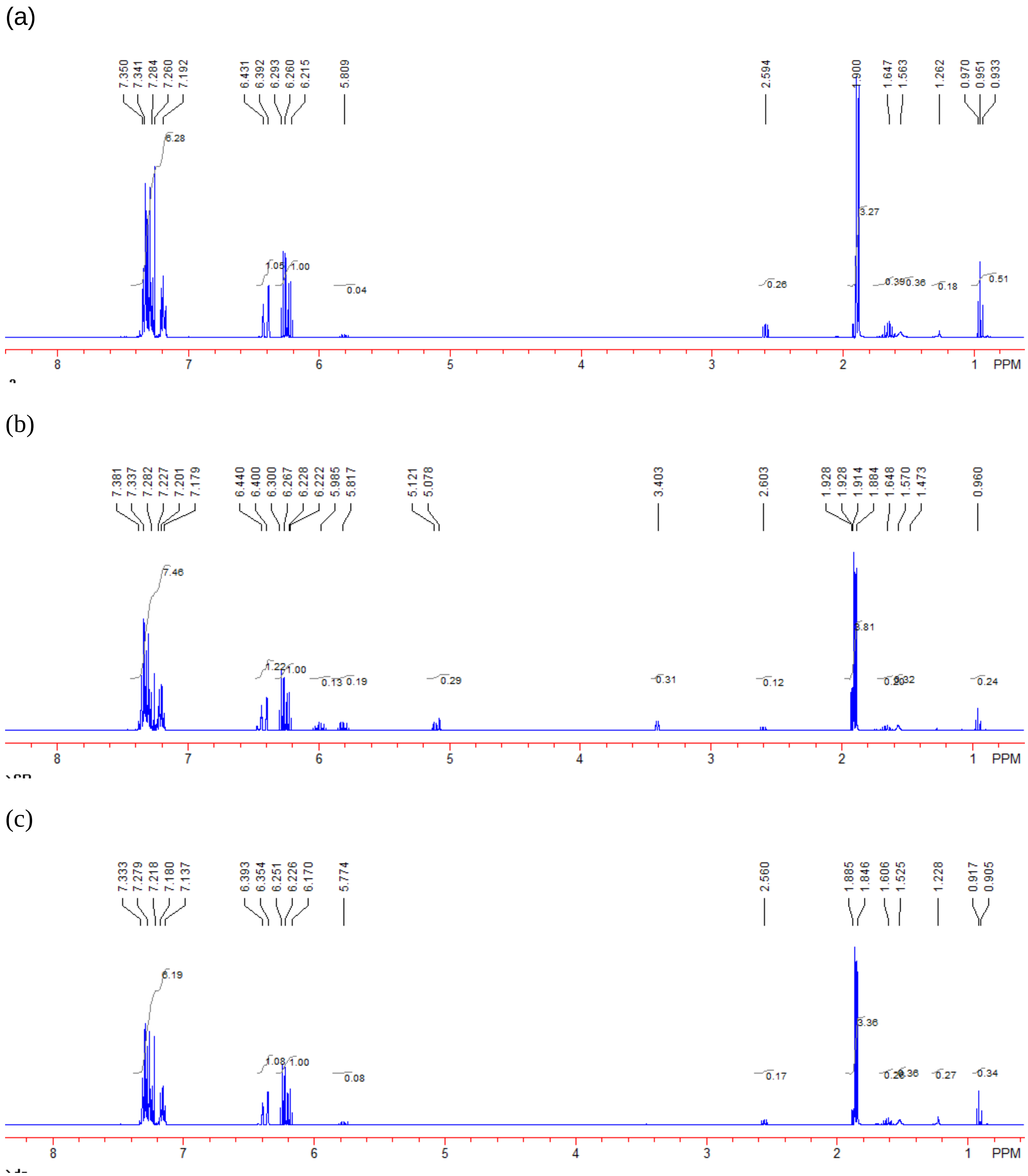

Figure S7. Proton NMR spectra obtained after reacting allylbenzene in $CDCl_3$ under H_2 conditions after 24 h with (a) C5 PdNP, (b) C5$_\beta$ PdNP, and (c) C5$_\gamma$ PdNP. The peaks at ~7.35-7.26 ppm, ~6.43-6.21 ppm, and 1.90 ppm are the characteristic shifts of 1-propenylbenzene. The peaks at ~7.19 ppm, ~1.90 ppm, ~1.65-1.56 ppm, and ~0.95 ppm are the characteristic shifts of 1-propylbenzene.
